# Multiple Sleep Alterations in Mice Lacking Cannabinoid Type 1 Receptors

**DOI:** 10.1371/journal.pone.0089432

**Published:** 2014-02-20

**Authors:** Alessandro Silvani, Chiara Berteotti, Stefano Bastianini, Viviana Lo Martire, Roberta Mazza, Uberto Pagotto, Carmelo Quarta, Giovanna Zoccoli

**Affiliations:** 1 PRISM lab, Department of Biomedical and Neuromotor Sciences, Alma Mater Studiorum – University of Bologna, Bologna, Italy; 2 Endocrinology Unit and Center for Applied Biomedical Research, Department of Medical and Surgical Sciences, S. Orsola University Hospital, Alma Mater Studiorum – University of Bologna, Bologna, Italy; INSERM, France

## Abstract

Cannabinoid type 1 (CB_1_) receptors are highly expressed in the brain and play a role in behavior control. Endogenous cannabinoid signaling is modulated by high-fat diet (HFD). We investigated the consequences of congenital lack of CB_1_ receptors on sleep in mice fed standard diet (SD) and HFD. CB_1_ cannabinoid receptor knock-out (KO) and wild-type (WT) mice were fed SD or HFD for 4 months (n = 9–10 per group). Mice were instrumented with electroencephalographic (EEG) and electromyographic electrodes. Recordings were performed during baseline (48 hours), sleep deprivation (gentle handling, 6 hours), sleep recovery (18 hours), and after cage switch (insomnia model paradigm, 6 hours). We found multiple significant effects of genotype on sleep. In particular, KO spent more time awake and less time in non-rapid-eye-movement sleep (NREMS) and rapid-eye-movement sleep (REMS) than WT during the dark (active) period but not during the light (rest) period, enhancing the day-night variation of wake-sleep amounts. KO had slower EEG theta rhythm during REMS. REMS homeostasis after sleep deprivation was less effective in KO than in WT. Finally, KO habituated more rapidly to the arousing effect of the cage-switch test than WT. We did not find any significant effects of diet or of diet x genotype interaction on sleep. The occurrence of multiple sleep alterations in KO indicates important roles of CB_1_ cannabinoid receptors in limiting arousal during the active period of the day, in sleep regulation, and in sleep EEG in mice.

## Introduction

The cannabinoid type 1 (CB_1_) receptor [1] is among the most widely expressed brain receptors [2] and plays a central role in the effects of cannabis-derived drugs of abuse [3]. CB_1_ receptors bind endogenous endocannabinoids such as arachidonoylethanolamide (anandamide) and 2-arachidonoylglycerol (2-AG) [4], which typically act as retrograde paracrine signals exerting negative feedback control of neuron firing [4], [5]. Endocannabinoids are tightly linked to metabolic control [6]: high-fat diet (HFD) modulates the endocannabinoid system in the brain [7], [8] and non-neural peripheral tissues [9]. Moreover, lack of CB_1_ receptors either in the whole body or only in neurons protects from adverse metabolic consequences of HFD [10]. Overactivation of the endocannabinoid system may thus contribute to the pathophysiology of obesity and the metabolic syndrome [6], [9]. However, modulation of CB_1_ receptor signaling also entails important consequences on behavior [4], [5]. This is dramatically highlighted by the recent failure of attempts to treat obesity and the metabolic syndrome with the CB_1_ receptor antagonist Rimonabant due to psychiatric side effects [11].

In previous work on rats, blockade of CB_1_ receptors at the beginning of the light period increased the time spent in wakefulness at the expense of NREMS and REMS [12]. Conversely, increase of endogenous anandamide levels increased the time spent in REMS and NREMS at the onset of the dark period only [13]. However, this effect was reversed only partially by a CB_1_ receptor antagonist and, thus, depended only marginally on CB_1_ receptors [13]. Similarly, the time spent in REMS was increased by intrahippocampal anandamide administration during the dark period [14]. However, anandamide administration repeated once a day for 15 days increased the time spent in REMS during both the light and the dark periods [15]. Acute CB_1_ receptor blockade impaired REMS recovery after deprivation, but NREMS homeostasis was not explored [16]. Furthermore, the effect of CB_1_ receptor blockade on sleep has not been assessed in conditions of HFD feeding. This would be of interest because enhanced CB_1_ receptor signaling in conditions of HFD feeding [7] may contribute to the increase in NREMS time observed in these conditions [17]. Moreover, HFD feeding changes endocannabinoid modulation of the activity of hypothalamic orexinergic neurons [18], which play a key role in energy homeostasis and wake-sleep state control [19].

While there is substantial evidence that CB_1_ receptors are involved in sleep regulation, several key questions still remain unanswered. It is still unclear whether CB_1_ receptors are equally important in controlling sleep amount at different times of day. This is of interest because CB_1_ receptors are expressed by and may modulate the activity of neurons in the hypothalamic suprachiasmatic nucleus, which make up the master body clock [20]. It is also unclear whether CB_1_ receptors are involved solely in REMS homeostasis or also in NREMS homeostasis, and whether sleep EEG also depends on CB_1_ receptor signaling. Finally, it is unknown whether CB_1_ receptor signaling plays a role in sleep alterations in conditions of HFD feeding or psychogenic insomnia. We aimed to address these questions by performing for the first time a complete sleep evaluation in CB_1_ receptor knock-out mice (KO) [21] fed either standard diet (SD) or HFD for 4 months and in their wild-type (WT) littermate controls.

## Methods

This study was carried out in accordance with the recommendations in the Guide for the Care and Use of Laboratory Animals of the National Institutes of Health. The protocol was approved by the Committees on the Ethics of Animal Experiments of the University of Bologna and of the Italian Ministry of Education, University, and Research (Permit Number: 8137). All surgery was performed under isoflurane anesthesia, and all efforts were made to minimize suffering.

### Mice

Experiments were performed on male KO mice with congenital deficiency of CB_1_ receptors [21] and their male WT littermate controls. KO mice were homozygous for the Cnr1tm1.1Ltz allele of the Cnr1 gene, which is characterized by deletion of the open reading frame [21]. All mice were congenic (7 generations of backcrossing) to C57Bl/6N, maintained in the laboratory animal facilities of the University of Bologna, Italy, and genotyped by polymerase-chain reaction as previously described [10], [21].

### Diets

Mouse breeders and weaned pups were fed a mouse SD (12.3 KJ/g: 11% from fat, 19% from proteins, 70% from carbohydrates; Dr. Piccioni Lab, Gessate, Milano, Italy) [10]. At 8 weeks of age, mice under study were randomly placed on HFD (18.9 KJ/g: 40% from fat, 15% from proteins, 45% from carbohydrates; Dr. Piccioni Lab) [10] or maintained on SD for >15 weeks until the termination of the experimental protocol, yielding 4 experimental groups: WT-SD (n = 9), KO-SD (n = 10), WT-HFD (n = 10), KO-HFD (n = 9).

### Recordings

Mice were instrumented under general anaesthesia (isoflurane 1.8–2.4% in O_2_) and intraoperative analgesic treatment (Carprofen 0.1 mg s.c., Pfizer Italy, Latina) as previously described [22]. In particular, mice were implanted with electrodes for frontal-parietal differential electroencephalographic (EEG) and electromyographic (EMG, neck muscles) recordings. Mouse age at surgery averaged 20.1±0.3 weeks (mean ± SEM) and did not differ significantly among groups. After surgery, mice were housed singly with gauze as nesting material to enrich environment and allowed 15–18 days of recovery and habituation to the recordings setup. Recordings were then performed on freely-behaving mice in their own cages as previously described [22]. EEG and EMG were transmitted via cable, and a rotating swivel (SL2+2C/SB, Plastics One, Roanoke, VA, USA) on a balanced suspensor arm prevented the cable from twisting and counterbalanced its weight, thus allowing unhindered movements to the mice. The EEG and EMG signals were amplified and filtered (EEG: 0.3–100 Hz with 50 Hz notch filter; EMG: 100–1000 Hz with 50 Hz notch filter; 7P511J amplifiers, Grass, West Warwick, RI, USA), digitized at 16-bit and 1024 Hz, and down-sampled at 128 Hz for data storage. Data acquisition was performed by means of custom software written in Labview (National Instruments) [22]. During recordings, mice were housed under a 12∶12-h light-dark cycle with ambient temperature set at 25°C and free access to water and food.

### Experimental Protocol

The experimental protocol consisted of the following sequence of recordings: baseline, sleep deprivation, sleep recovery, cage-switch test. Baseline recordings were performed for 48 hours on animals undisturbed in their own cages. Total sleep deprivation was performed by gentle handling [23] for 6 hours starting from lights on (Zeitgeber Time 0, ZT0) to ZT6, when sleep is most abundant and deep in mice [24]. Sleep recovery was assessed during undisturbed conditions for 18 hours from ZT6 to ZT24 [24]. Finally, recordings were performed during a cage-switch test [25] for 6 hours from ZT0 to ZT6. This mild psychological stress, which consists of exposing a male mouse to bedding soiled by other males, has been recently described as a paradigm of psychogenic insomnia in rats [26].

### Data Analysis

Data analysis was performed with MatLab (Mathworks, Natick, MA, USA). Scoring of wake-sleep states (wakefulness, NREMS, and REMS) was performed visually on the basis of raw EEG and EMG recordings with 4-s resolution as previously described in detail [25]. We estimated the daily variation in wake-sleep amounts by computing the absolute value of the difference in the time spent in each state between the dark and the active period and expressing it as a percentage of the total amount of wakefulness or sleep over 24 h. We then sought to determine whether potential differences in total 24-h wake-sleep amounts between groups of mice involved wake-sleep episodes of specific duration. To this aim, we partitioned the total recording time spent in each wake-sleep state over 48-h baseline recordings as a function of wake-sleep episode duration. The minimum episode duration was set at 12 s [25]. Values above this threshold were lumped into 4 time bins with thresholds set at 1, 2, and 3 minutes for sleep states and at 1, 5, and 30 minutes for wakefulness. These thresholds were set empirically to reflect the different distribution of the duration of wakefulness and sleep episodes. Spectral analysis of the EEG signal was performed on artifact-free 4-s epochs using discrete Fourier transform. For the purpose of comparing EEG power spectra during sleep, ancillary analyses were also performed on NREMS and REMS episodes with duration ≥32 s, on which we could apply the Fourier transform on longer (30 s vs. 4 s) EEG data windows to improve frequency range and resolution. Inter-individual differences in EEG spectral power were accounted for as follows. The EEG power spectral density during NREMS and REMS was expressed as a percentage of total EEG power in each state. EEG spectral power in the delta frequency range (1–4 Hz: slow-wave activity, SWA) during NREMS at baseline and during sleep recovery was expressed as a percentage of the mean SWA during NREMS at baseline between ZT8 and ZT12 [24].

### Statistics

Statistical tests were performed with SPSS (SPSS, Chicago, IL, USA) and significance at P<0.05 unless otherwise stated. Data were reported as means ± SEM and analysed by ANOVA (one-way ANOVA or GLM procedure with mixed-model design and Huynh-Feldt correction as appropriate). Differences between KO and WT were tested by *t*-tests with four planned comparisons (KO-SD vs. WT-SD, WT-HFD vs. WT-SD, KO-HFD vs. KO-SD, and KO-HFD vs. WT-HFD). The significance of these *t*-tests was set at the conventional *P*<0.05 level in case of significant group or interaction effect at one-way or mixed-model ANOVA, respectively, and with the false discovery rate procedure [27] otherwise. Correlations between REMS recovery and wake-sleep time at baseline were analysed with Pearson’s coefficient. Finally, in order to estimate the significance of the main effects of genotype and diet and of their interaction, we performed a two-way factorial ANOVA with genotype (2 levels: KO and WT) and diet (2 levels: SD and HFD) as factors.

## Results

### KO Mice had a Lower Body Weight than WT Irrespective of Diet

**Figure 1:**
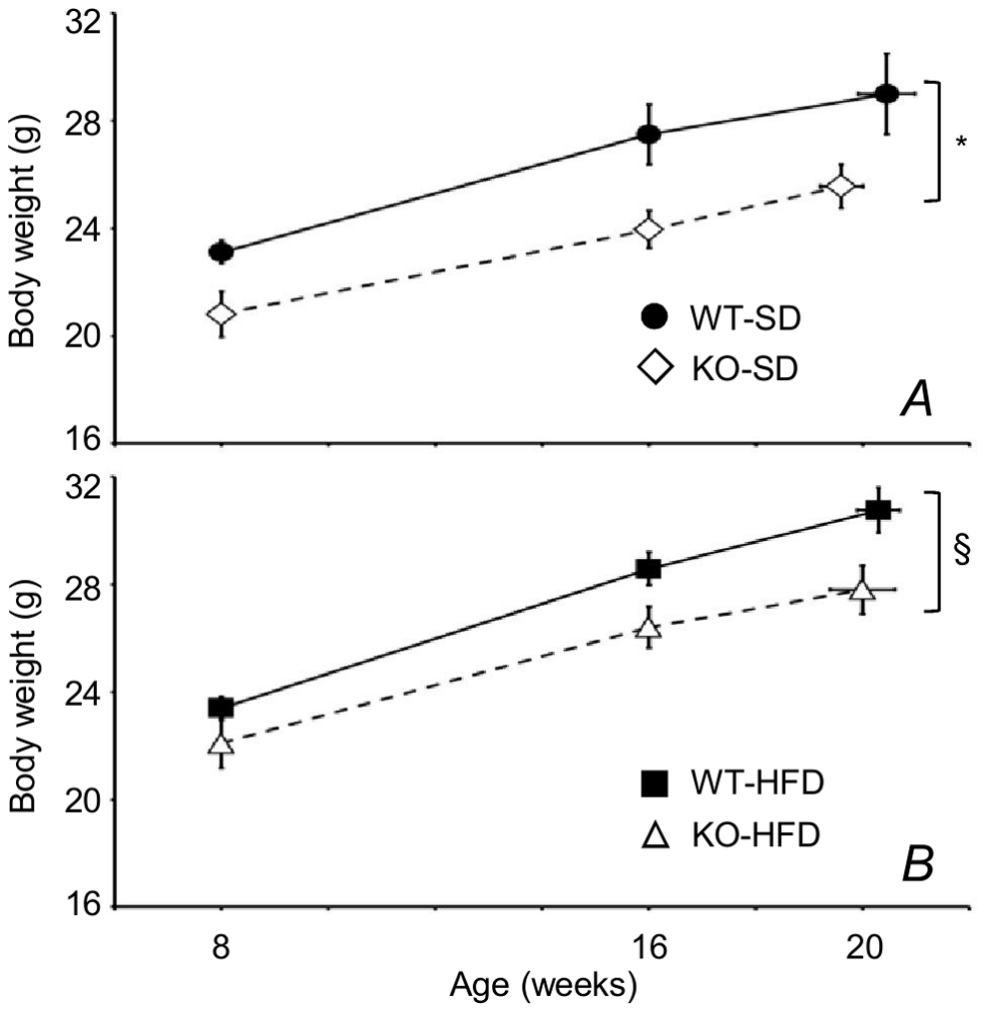
Body weight as a function of age from onset of dietary treatment to surgery in cannabinoid type 1 (CB_1_) receptor knock-out mice (KO) and wild-type (WT) mice. Fed standard diet (SD, A) or high-fat diet (HFD, B). Age of the mice at surgery is reported as the abscissa of the rightmost data points and did not differ significantly between groups (P = 0.219, ANOVA). Data are means ± SEM with n = 9–10 per group. * and §, P<0.05, ANOVA, main effect of genotype on mice fed SD and HFD, respectively.

The monitoring of body weight in mice maintained either on SD or HFD indicated that KO had a significantly lower body weight than WT controls (Figure 1). However, neither the percentage weight gain assessed after 2 months of diet nor the body length at autopsy differed significantly among groups of mice (Table S1).

### KO Mice Slept Less than WT during the Dark Period

**Figure 2:**
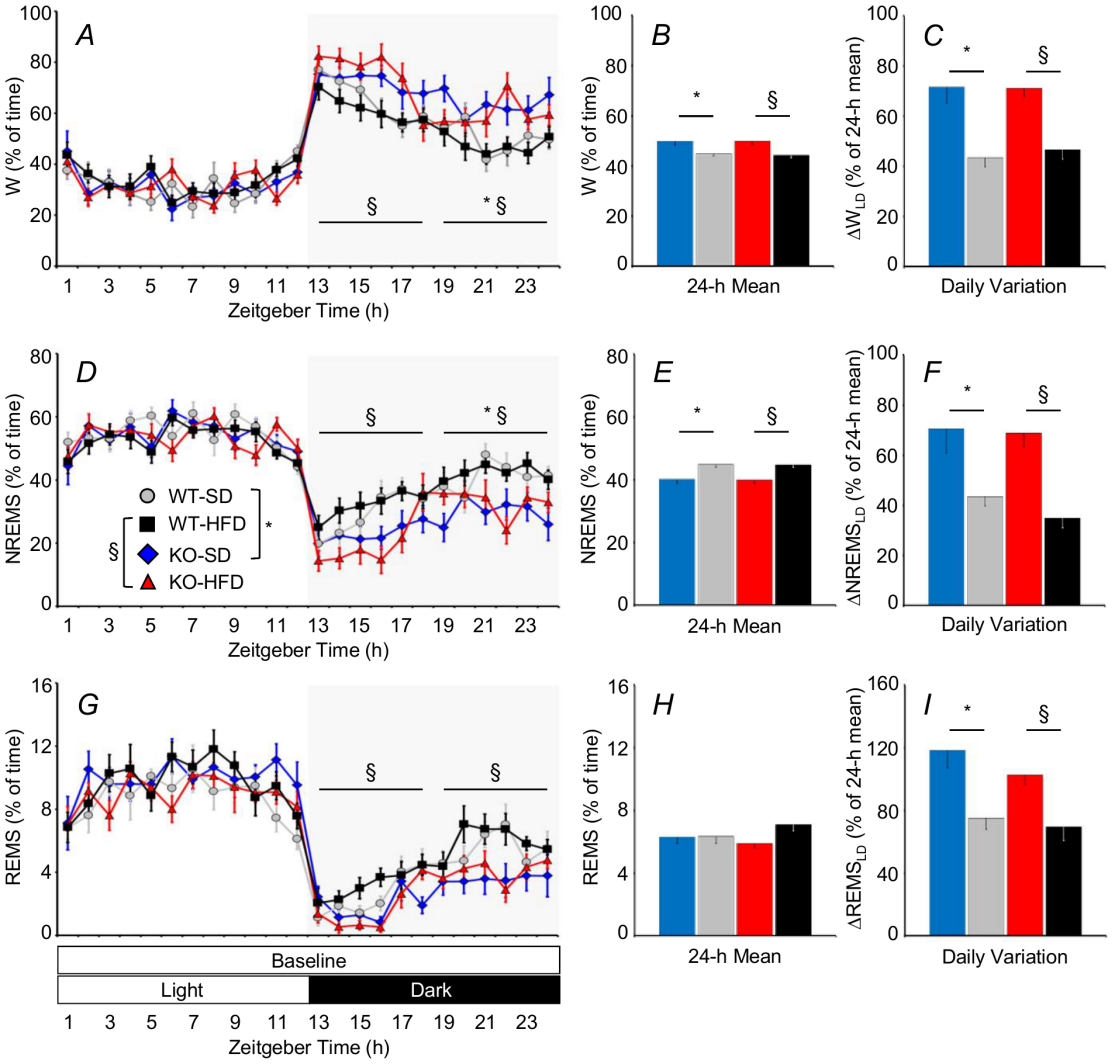
Percentage of baseline recording time spent in wakefulness (W), non-rapid-eye-movement sleep (NREMS), and rapid-eye-movement sleep (REMS) as a function of the time of day (A, D, and G) and as 24-hour average (B, E, and H). Panels C, F, and I show the difference in the amount of time spent in each wake-sleep state between the light and dark period expressed as a percentage of the 24-h amount of time spent in each state. Zeitgeber time is time from lights on. Statistical analysis in panels A, D, and G was performed on average values over 6-hour periods (horizontal lines). Data are means ± SEM in KO and WT fed SD or HFD, with n = 9–10 per group. * and §: P<0.05, WT-SD vs. KO-SD and WT-HFD vs. KO-HFD, respectively. Abbreviations have the same meaning as in Figure 1.

KO-SD spent less time in NREMS and more time awake than WT-SD at the end of the dark period (Figure 2A,D). In KO-HFD compared with WT-HFD, these alterations were significant during the whole dark period and were concomitant with a loss of REMS time (Figure 2A,D,G). In KO fed either diet, the changes in wake-sleep time during the dark period were not compensated during the light period, leading to an excess of wakefulness at the expense of NREMS over 24 hours (Figure 2B,E). In KO, this excess of wakefulness compared with WT was similar on either diet (SD, +70±25 minutes/24 h; HFD, +79±24 minutes/24 h). In KO, this excess of wakefulness amplified the daily variation of wake-sleep amounts between the light and dark periods compared with WT under either dietary treatment (Figure 2C,F,I). Conversely, differences between WT-HFD and WT-SD and between KO-HFD and KO-SD were not significant for any of the variables reported in Figure 2.

**Figure 3:**
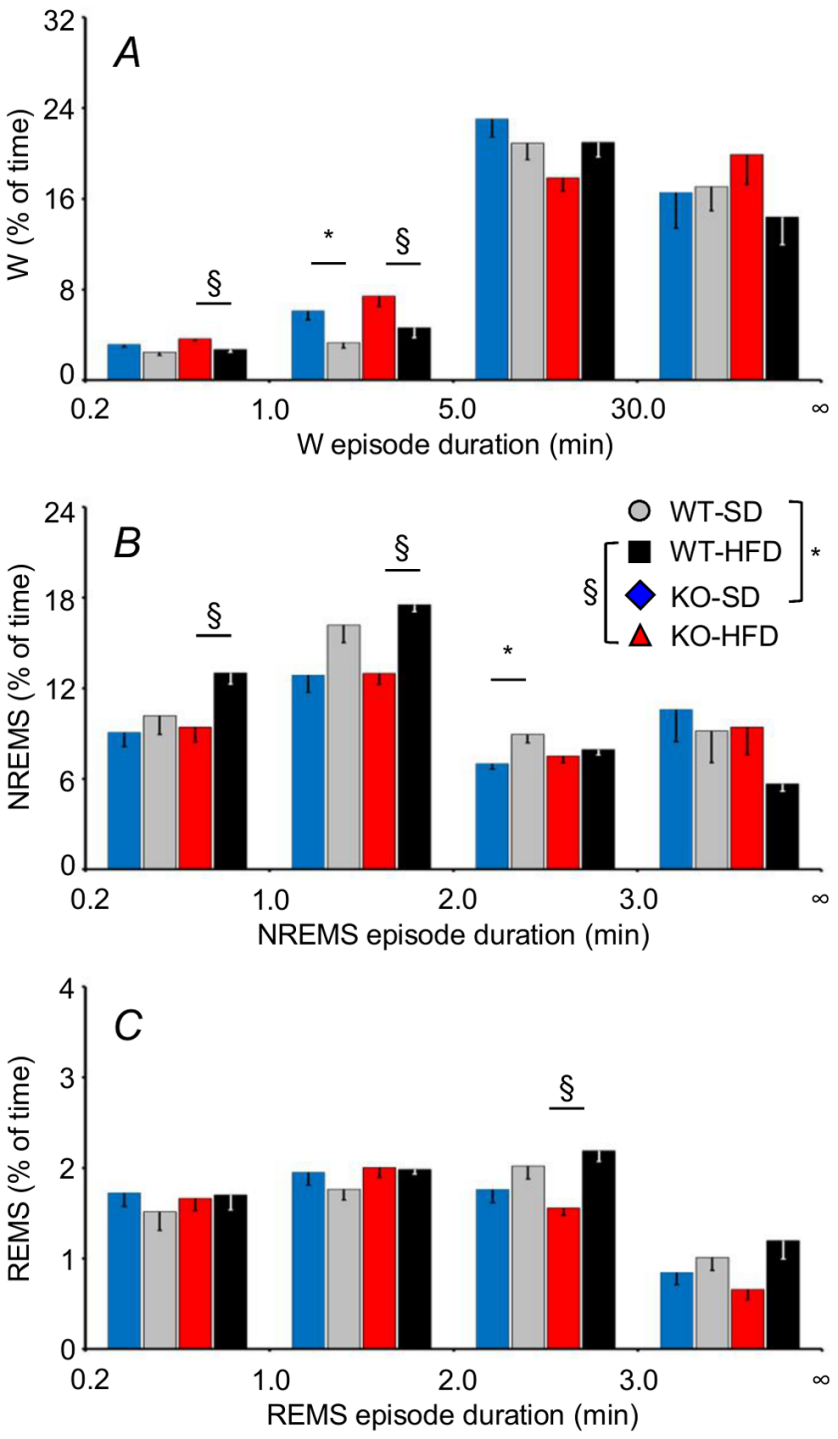
Percentage of baseline recording time spent in W, NREMS, and REMS as a function of episode duration. Data are means ± SEM in KO and WT fed SD or HFD, with n = 9–10 per group. Abbreviations and symbols have the same meaning as in Figure 2.

In KO-SD, the excess of wakefulness and the loss of NREMS over 24 hours compared with WT-SD preferentially concerned episodes with duration of 1–5 minutes and 2–3 minutes, respectively (Figure 3). Compared with WT-HFD, KO-HFD showed excess wakefulness in episodes shorter than 5 minutes, loss of NREMS in episodes shorter than 2 minutes, and loss of REMS in episodes with duration between 2 and 3 minutes. We did not find any significant difference concerning comparisons between WT-HFD and WT-SD and comparisons between KO-HFD and KO-SD.

### KO Mice showed Alterations in EEG Rhythms during Sleep

**Figure 4:**
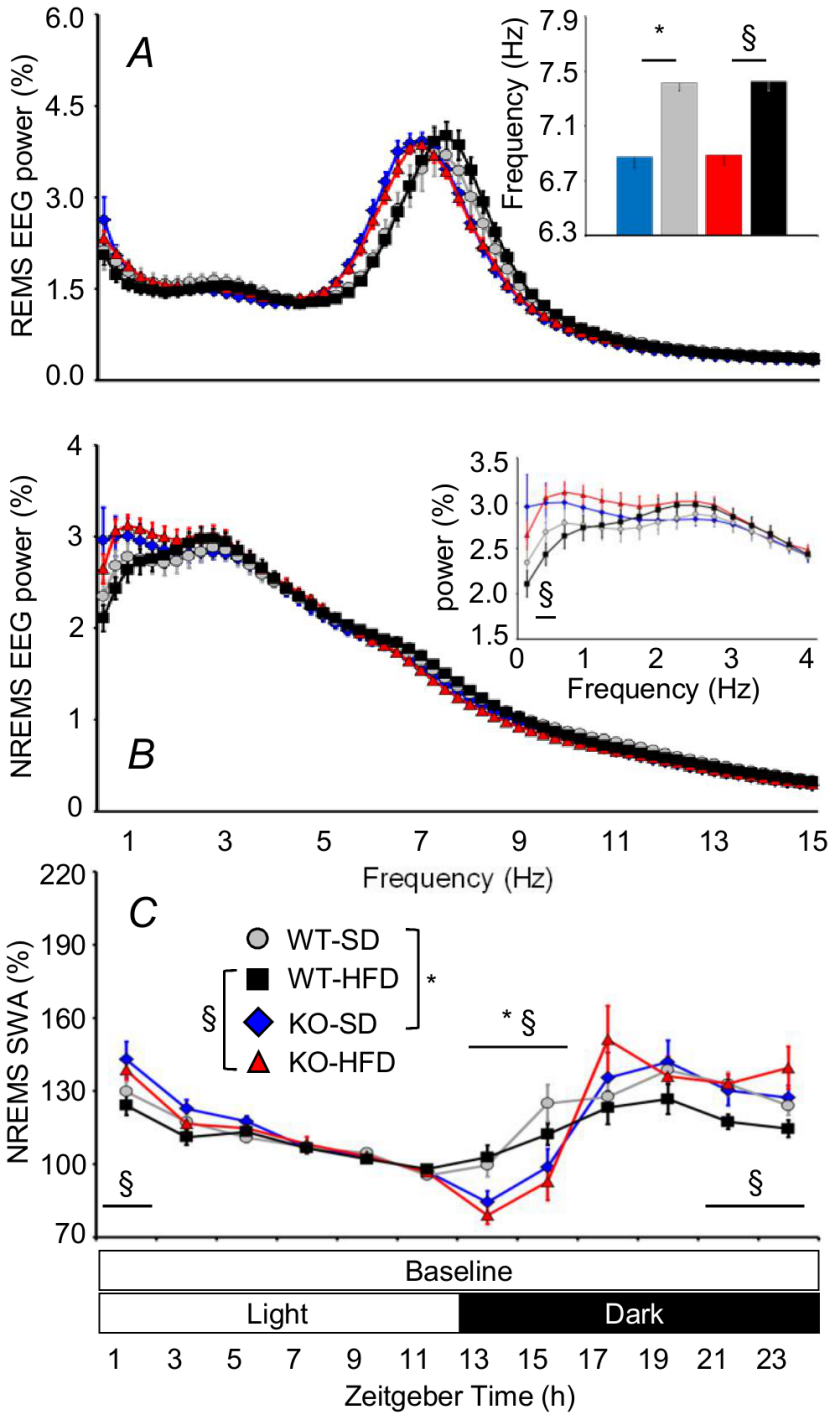
Electroencephalographic (EEG) rhythms during sleep in KO and WT mice. Panels A and B show EEG power spectral density in all REMS and NREMS epochs during the 48-hour baseline recordings expressed as a percentage of the respective total EEG spectral power. The inset in A shows the frequency of the EEG spectral peak. The inset in B shows magnification of NREMS spectral power at frequencies <4 Hz. Panel C shows power in the delta frequency range (1–4 Hz, EEG slow-wave activity, SWA) during NREMS in baseline conditions. EEG SWA was normalized to values in the last 4 hours of the light period. Data are means ± SEM in KO and WT fed SD or HFD, with n = 9–10 per group. Abbreviations and symbols have the same meaning as in Figure 2.

The EEG theta rhythm during REMS epochs in the 48-h baseline period was slower in KO than in WT irrespective of diet (Figure 4A). This difference persisted in ancillary analyses performed on longer (30 s vs. 4 s) EEG data windows to increase frequency resolution (data not shown) and occurred during the light as well as during the dark period (Figure S1).

EEG spectral power during NREMS epochs in the 48-h baseline period was also greater in KO-HFD than in WT-HFD at 0.5 Hz (Figure 4B). This difference was confirmed at frequencies 0.53–0.86 Hz in ancillary analyses performed on longer (30 s vs. 4 s) EEG data windows to increase frequency resolution (data not shown), and persisted during the light period, but not during the dark period (Figure S2). KO showed lower EEG SWA during NREMS at the onset of the dark period irrespective of diet (Figure 4C). KO-HFD also showed higher EEG SWA than WT-HFD during NREMS immediately before and after the dark-light transition.

We did not find any significant difference in the EEG spectral power during REMS and NREMS, including its SWA, between WT-HFD and WT-SD or between KO-HFD and KO-SD. The EEG spectral power during epochs of wakefulness in baseline recordings also did not differ significantly among groups of mice either during the light or during the dark period (Figure S3).

### KO Mice fed HFD had Impaired REMS Homeostasis

**Figure 5:**
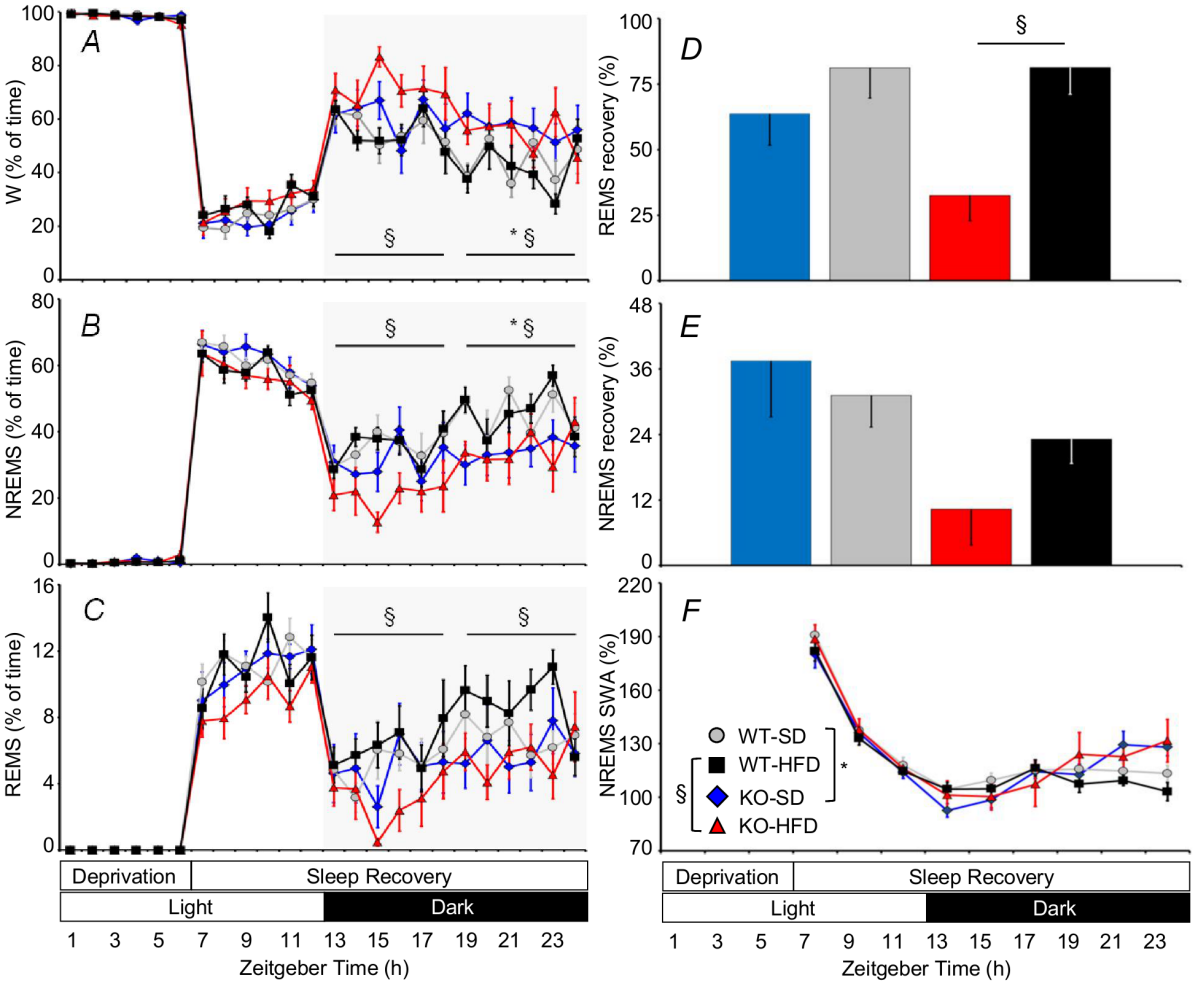
Wake-sleep profile during and after sleep deprivation in KO and WT mice. Panels A, B, and C show the percentage of recording time spent in W, NREMS, and REMS as a function of the time of day during sleep deprivation and subsequent sleep recovery. Statistical analysis was performed on average values over 6-hour periods (horizontal lines). Panels D and E show the percentage of NREMS and REMS time lost during sleep deprivation, respectively, which was recovered at the end of the sleep recovery period. Panel F shows EEG SWA in NREMS during recovery after sleep deprivation with the same scale as in Figure 3. Data are means ± SEM in KO and WT fed SD or HFD, with n = 9–10 per group. Abbreviations and symbols have the same meaning as in preceding figures.

Notwithstanding the increase in sleep pressure due to prior sleep deprivation, KO spent less time asleep and more time awake than WT during sleep recovery in the dark (Figure 5A–C), in agreement with findings during baseline recordings (Figure 2A,D,G). After 18 hours of sleep recovery, WT had recovered approximately 80% of the REMS time lost during sleep deprivation irrespective of diet (Figure 5D). REMS recovery was significantly less effective in KO-HFD (32%) than in WT-HFD, whereas it did not differ significantly between KO-SD and WT-SD. Taking into account all mice under study, mice showing the greater propensity to wakefulness during the dark period at baseline showed the worse REMS recovery (Pearson r = −0.33, P = 0.04, n = 38). Recovery of NREMS lost during sleep deprivation did not differ significantly among groups either in terms of time (Figure 5E) or intensity, the latter as judged from EEG SWA (Figure 5F). Differences in EEG SWA did not occur among mice during NREMS with increased sleep pressure during sleep recovery. Similarly, we did not find any significant difference between WT-HFD and WT-SD or between KO-HFD and KO-SD in sleep homeostasis after sleep deprivation.

### KO Mice Habituated more Rapidly than WT to the Arousing Effect of the Cage-switch Test

**Figure 6:**
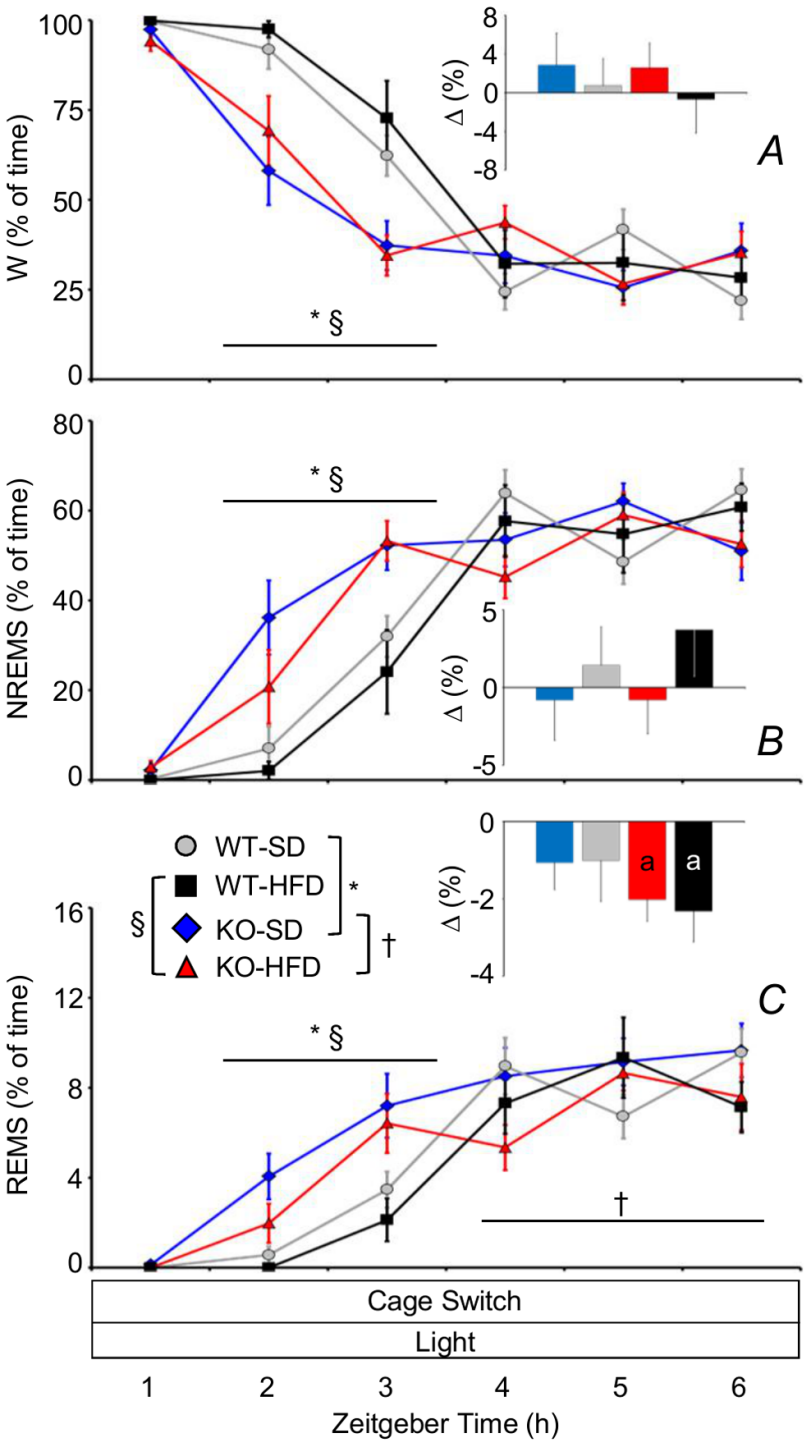
Wake-sleep profile during 6 h cage-switch test in KO and WT mice. Percentage of recording time spent in W (A), NREMS (B), and REMS (C) as a function of the time during a cage-switch test. Statistical analysis was performed on the first hour, the average of the second and third hours (horizontal lines), and the average of the last three hours of the cage-switch test. Insets show differences (Δ) in the percentage of recording time spent in a given wake-sleep state between the last 3 hours of the cage-switch test and the corresponding time period during baseline recordings. Data are means ± SEM in KO and WT fed SD or HFD, with n = 9–10 per group. a, P<0.05 vs. baseline recordings. †, P<0.05, KO-HFD vs. KO-SD. Other abbreviations and symbols have the same meaning as in preceding figures.

Mice spent most of the first hour of the cage-switch test awake irrespective of genotype and diet (Figure 6). For the subsequent 2 hours, vigilance decreased progressively, and this process was significantly more rapid in KO than in WT irrespective of diet. During the last 3 hours of the cage-switch test, the percentage of time spent in wakefulness and NREMS did not differ among groups, whereas the percentage of time spent in REMS was lower in KO-HFD than in KO-SD. In this last period, KO and WT fed HFD spent less time in REMS than they did at the same ZT during baseline recordings (Figure 6C, inset). With this exception, the time spent in each wake-sleep state did not differ during the last 3 hours of cage-switch test compared with baseline conditions at the same ZT (Figure 6, insets). No significant differences in the arousing effect of the cage switch test were apparent between WT-HFD and WT-SD.

### Partitioning the Effects of Cnr1 KO Genotype, Diet, and Genotype x Diet Interaction on Sleep and Sleep EEG

A factorial ANOVA with diet (SD vs. HFD) and genotype (KO vs. WT) as factors showed that without exception, the genotype factor was significant for all of the variables that differed significantly between KO-SD and WT-SD and/or between KO-HFD and WT-HFD. Conversely, we did not find any statistical significance of the main effect of diet and of the interaction effect between diet and genotype.

## Discussion

Our study yielded four main findings. KO-SD and KO-HFD spent more time in wakefulness during the dark period than WT-SD and WT-HFD, respectively, thereby enhancing their daily variation in wake-sleep amounts between the dark (active) and the light (rest) periods (Figure 2). KO-SD and KO-HFD showed a slower EEG theta rhythm in REMS than WT-SD and WT-HFD, respectively (Figure 4). REMS homeostasis was less effective in KO-HFD than in WT-HFD (Figure 5). The increase in vigilance entailed by the cage-switch test lasted less in KO-SD and KO-HFD than in WT-SD and WT-HFD, respectively (Figure 6).

We found a greater occurrence of statistically significant differences in sleep regulation and sleep EEG between KO-HFD and WT-HFD than between KO-SD and WT-SD. This may reflect insufficient statistical power of the KO-SD vs. WT-SD comparison and/or a genuine enhancement of the effect of genotype (i.e., Cnr1 gene mutation in KO) by diet. To clarify this, we run a dedicated factorial ANOVA statistics, which invariably indicated significance of the main effect of genotype, but no significance of either the diet x genotype interaction effect or the main effect of diet. The significance of the main effect of genotype supports the view that the CB_1_ receptor deficiency caused by the Cnr1 gene mutation has a significant impact on sleep and sleep EEG across dietary conditions. Accordingly, differences in sample means between KO-SD and WT-SD had the same direction as those between KO-HFD and WT-HFD even when only the latter resulted statistically significant. Conversely, non-significance of the diet x genotype interaction effect neither supports nor excludes the hypothesis that the effect of Cnr1 mutation is enhanced by HFD. This hypothesis thus stays open, and would be worthwhile to test with greater statistical power because of increased sample size, sequential administration of SD and HFD to the same subjects, and administration of more powerful dietary challenges.

The lack of significant main effects of diet in the present study may be due to the fact that we chose to administer a mild HFD regimen (i.e., 40% calories from fat) for an extended period of time (i.e., ≥15 weeks) starting at an early age (i.e., at 8 weeks of age, which is around puberty in mice). Conversely, previous findings indicate that 6 weeks of nutritionally extreme HFD (i.e., 60% calories from fat) administered to fully developed mice (i.e., at 6 months of age) increase NREMS at the expense of wakefulness [17]. We may have failed to replicate these findings because our HFD was too mild and/or because our early-onset long-term treatment made mice tolerant to the effects of HFD. Regardless, we have previously shown that the same dietary protocol as in the present study increases plasma leptin, insulin, glucose, free fatty acids, triglycerides, and total cholesterol in WT mice [10].

Our study expanded results of previous pharmacological studies [12]–[16] to conditions of life-long lack of CB_1_ receptor signaling, which allowed us to rigorously assess sleep amounts at different times of day and to assess sleep homeostasis without the confounding effects associated with the pharmacokinetics of CB_1_ receptor agonists or antagonists. The present results thus contribute a critical piece of evidence to the demonstration that CB_1_ receptor signaling limits arousal during the active part of the day. In this respect, our finding that KO show increased behavioral arousal than WT only during the dark period (Figures 2A and 5A) fit well with our previous observations that these mice show hyperactivity of the hypothalamo-pituitary-adrenal axis only during the dark period [28]. Taken together, these studies provide the rationale for targeted chronobiological experiments to fully characterize the amplitude of free-running circadian rhythms in KO.

Our data support the view that CB_1_ receptors play a role in REMS homeostasis (Figure 5D), but not in NREMS homeostasis (Figure 5E,F). Moreover, our correlation analysis suggests that CB_1_ signaling impacts on REMS homeostasis at least in part by modulating the propensity to arousal during the dark period, which corresponded to the last two thirds of the sleep recovery period in our experimental protocol. Interestingly, although NREMS homeostasis after acute sleep deprivation was preserved in KO, loss of NREMS time during the dark period in KO was not compensated during the light period (Figure 2E). This latter finding indicates that CB_1_ receptor signaling contributes to set the long-term need of NREMS time. On the other hand, differences between KO and WT in the total time spent in wakefulness and NREMS over 24 hours concerned relatively short episodes of wakefulness and NREMS (Figure 3C,F). This suggests that CB_1_ receptors are needed to maintain the physiological balance between wakefulness and NREMS during periods of drowsiness and unstable sleep.

Our findings that chronic lack of CB_1_ receptors alters EEG rhythms during sleep (Figure 4A,B) are to our knowledge entirely new, and we have not yet explored their detailed mechanisms and their functional implications. In rodents, EEG theta rhythm during REMS largely results from volume conduction from the hippocampus and is driven by a circuitry involving the medial septum and the diagonal band of Broca [29]. CB_1_ receptors are expressed in the medial septum and hippocampus, [2], [30], where electrophysiological experiments indicates that they modulate theta rhythm by changing the temporal coordination of cell assemblies [31]. The hippocampal theta oscillation may bring together in time the activity of sensory- and memory-activated neurons [29], thus impacting on memory performance [31]. The changes in EEG theta rhythm that we found in KO (Figure 4A and Figure S1) may thus underlie, at least in part, the effect of CB_1_ receptors on memory encoding [31], [32]. Interestingly, the slight changes that we found in KO in terms of NREMS EEG power (Figure 4B and Figure S2) were in the frequency range (<1 Hz) of the so-called slow oscillation, which results from the interplay between a synaptically-based cortical oscillator and intrinsic thalamic oscillators, and which has also been linked to memory processes [33]. However, this result should be considered preliminary until replicated with lower cutoff frequency of the EEG high-pass filter, which was 0.3 Hz in this study, and with specific algorithms for the assessment of the EEG slow oscillation in the time and frequency domains.

The cage-switch test has been proposed as a paradigm of insomnia induced by internally generated psychological stress on rats [26]. On this basis, our findings during the second and third hours of the cage-switch test (Figure 6) suggest that CB_1_ receptors are necessary to sustain vigilance and inhibit sleep in conditions of internally generated psychological stress. A prolonged response of the hypothalamus-pituitary-adrenal axis to restraint stress has been reported in KO mice [34]. However, reports on changes in anxiety and motility have been inconsistent on different strains of KO mice lacking CB_1_ receptors [21], [35], [36] and yielded non-significant results in the specific strain we studied [21]. We found that after the fourth hour of the cage-switch test, wake-sleep time had returned to the levels measured during previous baseline recordings at the same ZT in most groups of mice. Conversely, previous experiments showed that during the last 2 hours of the cage-switch test, rats still spent more time in wakefulness and co-activated subcortical structures involved in both wakefulness and sleep compared with rats switched to a cage with clean bedding [26]. A robust insomnia protocol may be expected not only to override sleep need, but also to reduce absolute sleep time compared with undisturbed (“healthy”) baseline conditions. We thus suggest that the first 3 hours of the cage-switch test may be more robust as a paradigm of insomnia than the last part of the test in mice.

While experiments on genetically-modified mice, such as those of the present study, offer distinct advantages over pharmacological approaches, they also suffer from well-known limitations. In particular, we studied mice lacking CB_1_ receptors in the whole body rather than solely in neurons. In line of principle, the effects that we observed may thus have been caused indirectly by peripheral changes reverberating onto the central nervous system. Experiments on mice with neuron-specific loss of CB_1_ receptors [37] may shed light on this issue. Moreover, the phenotype of KO may reflect at least in part compensatory mechanisms to the congenital deficiency of CB_1_ receptors, possibly arising during early prenatal and postnatal development. Accordingly, careful studies on neural connectivity in KO have revealed slight differences compared with WT [38]. Thus, CB_1_ receptors may play a necessary role in adult sleep control by shaping early neural development.

In conclusion, our data indicate that lifelong lack of CB_1_ receptors causes significant alterations in sleep regulation and in sleep EEG. Our data implicate CB_1_ receptors in a wide array of sleep-related functions, which include the short-term REMS homeostasis, the balance between wakefulness and NREMS during periods of unstable sleep, the propensity to arousal during the active period of the day and in conditions of situational insomnia, and the frequency of sleep EEG rhythms. In perspective, these data raise the hypothesis that sleep alterations may be elicited by long-term blockade of CB_1_ receptors to treat obesity.

## Supporting Information

Figure S1 Electroencephalographic (EEG) power spectral density during rapid-eye-movement sleep (REMS) in cannabinoid type 1 (CB1) receptor knock-out mice (KO) and wild type (WT) mice. Panels A and B show EEG power spectral density in all epochs of REMS during the light and dark periods of the 48-hour baseline recordings, respectively, expressed as a percentage of the respective total EEG spectral power. The insets show the frequency of the EEG spectral peak. Data are means ± SEM in KO and WT mice fed standard diet (SD) or high-fat diet (HFD), with n = 9–10 per group. * and §: P<0.05, WT-SD vs. KO-SD and WT-HFD vs. KO-HFD, respectively.
(TIF)

Figure S2 EEG power spectral density during non-rapid-eye-movement sleep (NREMS) in KO and WT mice. Panels A and B show EEG power spectral density in all epochs of NREMS during the light and dark periods of the 48-hour baseline recordings, respectively, expressed as a percentage of the respective total EEG spectral power. The insets show magnification of NREMS spectral power at frequencies <4 Hz. Data are means ± SEM in KO and WT fed SD or HFD, with n = 9–10 per group. Abbreviations and symbols have the same meaning as in Figure S1.
(TIF)

Figure S3 EEG power spectral density during wakefulness (W) in KO and WT mice. Panels A and B show EEG power spectral density in all epochs of W during the light and dark periods of the 48-hour baseline recordings, respectively, expressed as a percentage of the respective total EEG spectral power. Data are means ± SEM in KO and WT fed SD or HFD, with n = 9–10 per group. Abbreviations have the same meaning as in Figure S1.
(TIF)

Table S1 Weight gain and body length of the mice studied.
(DOCX)
