# Supplementary figures and images for: Multiple Sleep Alterations in Mice Lacking Cannabinoid Type 1 Receptors
Source: PLoS One. 2014 Feb 20;9(2):e89432. doi: 10.1371/journal.pone.0089432 (PMC3930731; doi:10.1371/journal.pone.0089432)

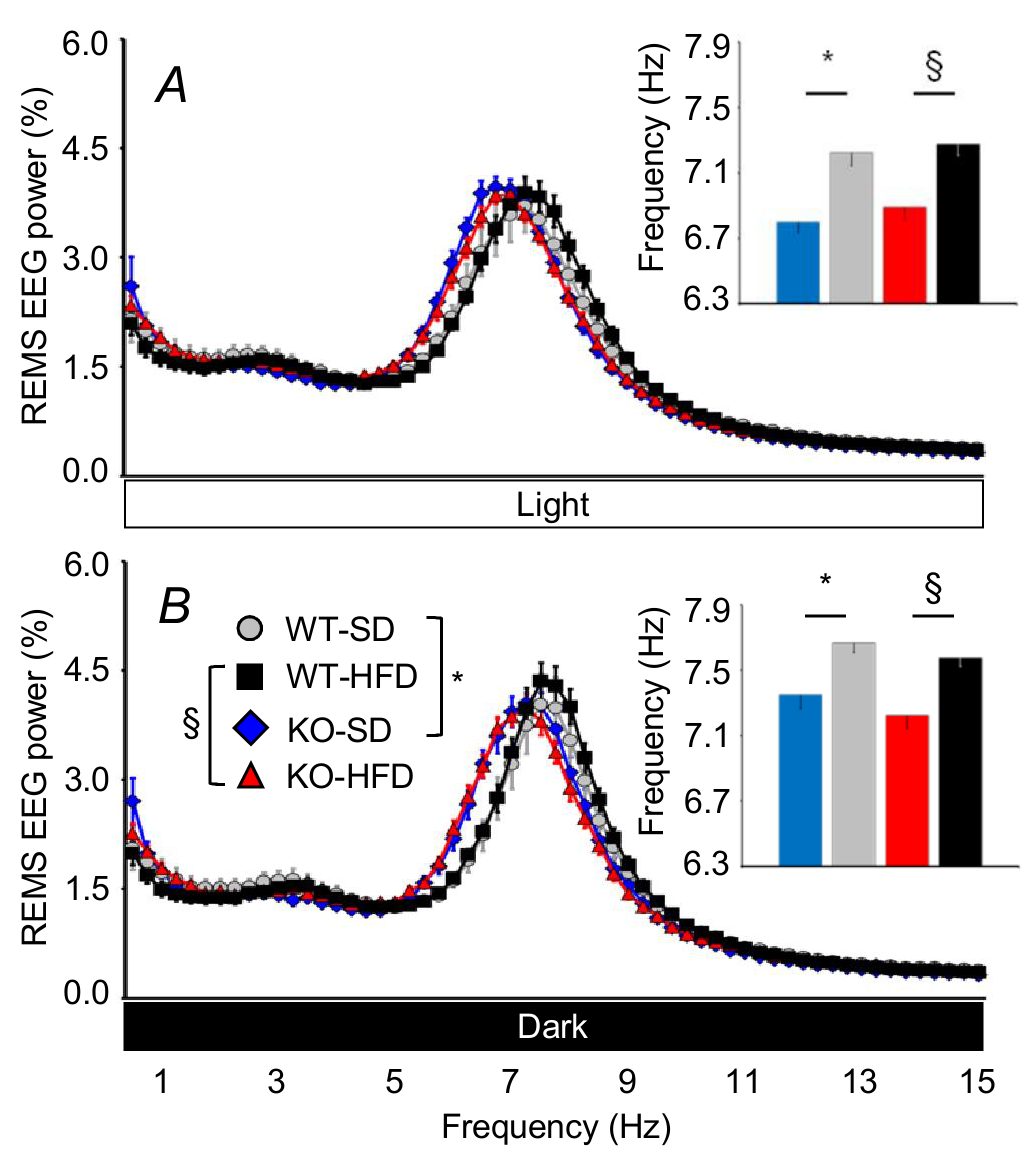

Supplement: Figure S1 — Electroencephalographic (EEG) power spectral density during rapid-eye-movement sleep (REMS) in cannabinoid type 1 (CB1) receptor knock-out mice (KO) and wild type (WT) mice. Panels A and B show EEG power spectral density in all epochs of REMS during the light and dark periods of the 48-hour baseline recordings, respectively, expressed as a percentage of the respective total EEG spectral power. The insets show the frequency of the EEG spectral peak. Data are means ± SEM in KO and WT mice fed standard diet (SD) or high-fat diet (HFD), with n = 9–10 per group. * and §: P<0.05, WT-SD vs. KO-SD and WT-HFD vs. KO-HFD, respectively. (TIF) [file pone.0089432.s001.tif]

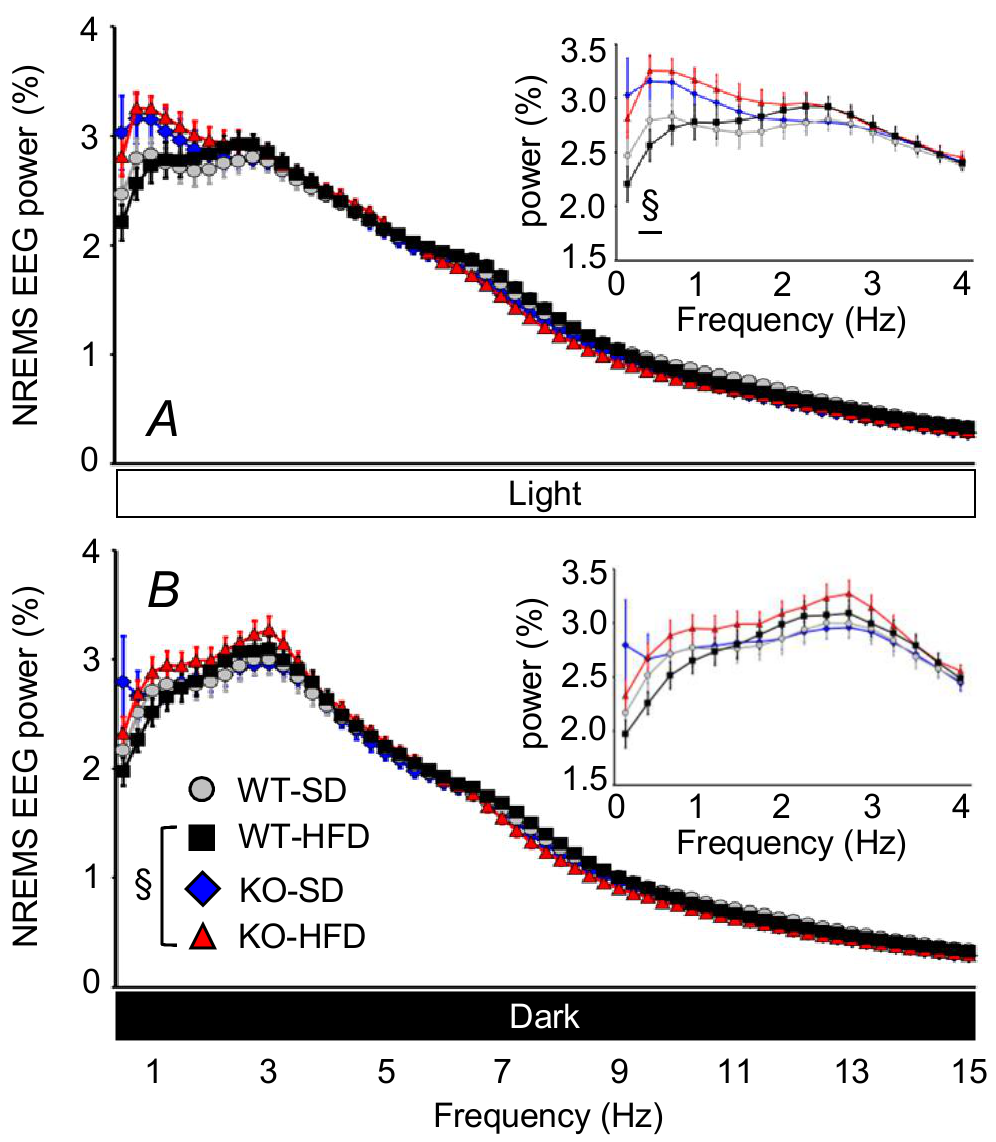

Supplement: Figure S2 — EEG power spectral density during non-rapid-eye-movement sleep (NREMS) in KO and WT mice. Panels A and B show EEG power spectral density in all epochs of NREMS during the light and dark periods of the 48-hour baseline recordings, respectively, expressed as a percentage of the respective total EEG spectral power. The insets show magnification of NREMS spectral power at frequencies <4 Hz. Data are means ± SEM in KO and WT fed SD or HFD, with n = 9–10 per group. Abbreviations and symbols have the same meaning as in Figure S1. (TIF) [file pone.0089432.s002.tif]

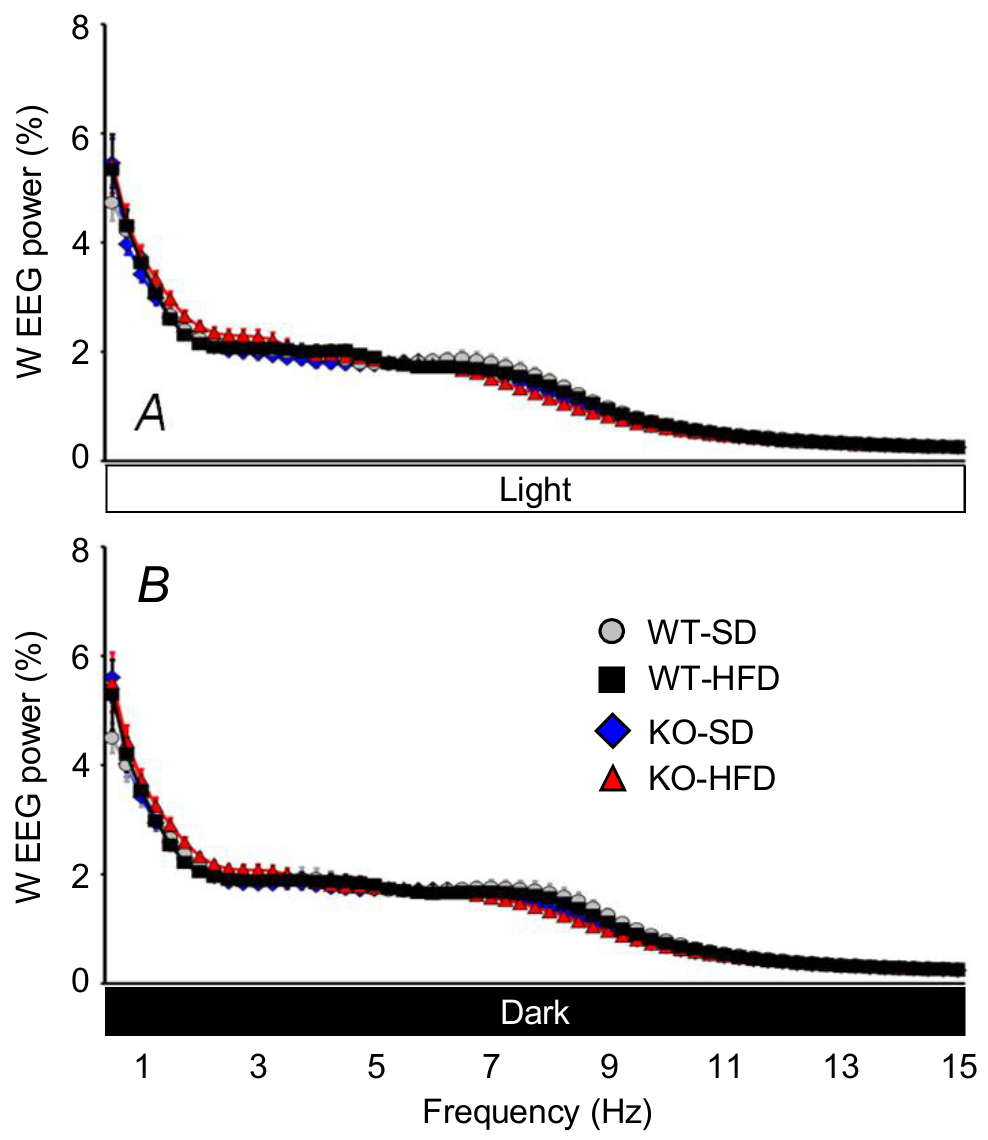

Supplement: Figure S3 — EEG power spectral density during wakefulness (W) in KO and WT mice. Panels A and B show EEG power spectral density in all epochs of W during the light and dark periods of the 48-hour baseline recordings, respectively, expressed as a percentage of the respective total EEG spectral power. Data are means ± SEM in KO and WT fed SD or HFD, with n = 9–10 per group. Abbreviations have the same meaning as in Figure S1. (TIF) [file pone.0089432.s003.tif]
