# Supplementary material for: Multiple Sleep Alterations in Mice Lacking Cannabinoid Type 1 Receptors
Source: PLoS One. 2014 Feb 20;9(2):e89432. doi: 10.1371/journal.pone.0089432 (PMC3930731; doi:10.1371/journal.pone.0089432)
Supplement: Table S1 — Weight gain and body length of the mice studied. (DOCX) [file pone.0089432.s004.docx]

**Table S1. Weight gain and body length of the mice studied**

|  | **WT** | | **KO** | |
| --- | --- | --- | --- | --- |
|  | **SD** | **HFD** | **SD** | **HFD** |
| **Weight gain (%)** | 19.0 ± 4.4 | 22.4 ± 2.9 | 15.7 ± 2.2 | 20.1 ± 2.5 |
| **Length (mm)** | 88 ± 1 | 89 ± 1 | 86 ± 2 | 86 ± 2 |

Weight gain, increase in body weight from the 8^th^ to the 16^th^ week of age expressed as a percentage of initial body weight. The length of the mice was measured from the anus to the snout at autopsy. Data are mean ± SEM in cannabinoid type 1 (CB_1_) receptor knock-out mice (KO) and wild-type (WT) mice fed standard diet (SD) or high-fat diet (HFD), with n = 9-10 per group.
